# A global chromoblastomycosis strategy and development of the global chromoblastomycosis working group

**DOI:** 10.1371/journal.pntd.0012562

**Published:** 2024-10-15

**Authors:** Dallas J. Smith, Flávio Queiroz-Telles, Fahafahantsoa Rapelanoro Rabenja, Roderick Hay, Alexandro Bonifaz, Marlous L. Grijsen, Romain Blaizot, Fernando Messina, Yinggai Song, Shawn R. Lockhart, Alexander Jordan, Alyson M. Cavanaugh, Anastasia P. Litvintseva, Tom Chiller, Marco Schito, Sybren de Hoog, Vania Aparecida Vicente, Muriel Cornet, Daniel Argaw Dagne, Lala S. Ramarozatovo, Conceição de Maria Pedrozo e Silva de Azevedo, Daniel Wagner C. L. Santos

**Affiliations:** 1 Mycotic Diseases Branch, Centers for Disease Control and Prevention, Atlanta, Georgia, United States of America; 2 Department of Public Health, Federal University of Paraná, Curitiba, Brazil; 3 LARTIC Laboratory, Faculty of Medicine, University of Antananarivo, Antananarivo, Madagascar; 4 King’s College London, London, United Kingdom; 5 Servicio de Dermatología, Hospital General de México “Dr. Eduardo Liceaga”, Mexico City, Mexico; 6 Oxford University Clinical Research Unit Indonesia, Faculty of Medicine Universitas Indonesia, Jakarta, Indonesia; 7 Centre for Tropical Medicine and Global Health, Nuffield Department of Medicine, University of Oxford, United Kingdom; 8 Department of Dermatology, Andrée Rosemon Hospital, 97306 Cayenne, French Guiana; 9 Tropical Biome and Immunophysiopathology (TBIP), Université de Lille, CNRS, Inserm, Institut Pasteur de Lille, U1019-UMR9017-CIIL-Centre d’Infection et d’Immunité de Lille, Centre Hospitalier de Cayenne, Université de Guyane, Cayenne, French Guiana; 10 Unidad Micología, Hospital de Enfermedades Infecciosas Francisco Javier Muñiz, Buenos Aires, Argentina, Hospital de Enfermedades Infecciosas Francisco Javier Muñiz, Buenos Aires, Argentina; 11 Department of Dermatology, Peking University First Hospital, Peking University, Beijing China; 12 Critical Path Institute, Tucson, Arizona, United States of America; 13 Radboudumc/CWZ Center of Expertise in Mycology, Nijmegen, the Netherlands; 14 Basic Pathology Department, Federal University of Parana, Curitiba, Brazil; 15 Université Grenoble Alpes, Grenoble, France; 16 Department of Control of Neglected Tropical Diseases, WHO, Geneva, Switzerland; 17 Hôpital Universitaire Joseph Raseta Befelatanana, Antananarivo, Madagascar; 18 Centre Hospitalier Universitaire de Befelatanana, Antananarivo, Madagascar; 19 Department of Medicine, Federal University of Maranhão, São Luís, Maranhão, Brazil; 20 Post-graduation Program of Health Science, Federal University of Maranhão, São Luís, Maranhão, Brazil; 21 Department of Infectious Diseases and Infection Control, Universidade Federal do Maranhão, Maranhão, Brazil; 22 Instituto D´Or de Pesquisa e Ensino, IDOR, Brazil; Gulu University, UGANDA

## Abstract

Chromoblastomycosis, an implantation mycosis, is a neglected tropical disease that causes decreased quality of life, stigma, and disability. The global burden of disease is unknown and data on disease epidemiology and outcomes are severely limited by a lack of access to needed diagnostic tools and therapeutics. The World Health Organization outlined targets for chromoblastomycosis in the Road Map for Neglected Tropical Diseases 2021–2030, but little progress has been made in initiating and implementing an effective control program globally. This lack of guiding policy and progress led to the recent formation of a Global Chromoblastomycosis Working Group which has developed a global chromoblastomycosis strategy. We describe this strategy, which outlines specific steps needed to improve technical progress, strategy and service delivery, and enablers. Clinicians, researchers, public and government officials, patients, and policy makers can align their time, expertise, and resources to improve the lives of communities affected by chromoblastomycosis through this strategy.

## Introduction

In 2017, the World Health Organization (WHO) designated chromoblastomycosis, an implantation mycosis (fungal disease) as a neglected tropical disease (NTD) [1]. Chromoblastomycosis manifests as visible changes to the skin, thus, is classified as a skin-NTD. Chromoblastomycosis is found primarily among poor agricultural workers in tropical and subtropical regions [2]. This fungal disease is caused by melanized, dematiaceous or black fungi; *Fonsecaea pedrosoi*, *F*. *monophora*, *F*. *nubica*, *Cladophialophora carrionii*, *Rhinocladiella aquaspersa*, and *Phialophora verrucosa* are the most common causes of chromoblastomycosis, but species distribution varies by region and climatic conditions [3,4]. Reliable disease burden estimates are lacking due to absence of national, regional, or global disease surveillance system or network. However, some researchers estimate the global burden to be greater than 10,000 cases [3,5]. Chromoblastomycosis is usually limited to the skin and subcutaneous tissue and is curable with antifungal therapy or non-pharmacological therapy when diagnosed early [6]. However, time to diagnosis is often prolonged in low- and middle-income countries (LMICs), resulting in lesions that are large, disabling, and refractory to treatment [7]. Rarely, certain causative species may lead to dissemination to the brain and can result in death [8]. Diagnosis requires trained personnel and laboratory capacity, which are often lacking in underserved areas. While chromoblastomycosis is considered a slow-progressing infection, delays in diagnosis and treatment in LMICs often result in patients presenting with large, purulent, and fibrotic lesions, which can lead to stigma and disability [9]. Chronic secondary bacterial coinfection can occur with severe lesions, and in some cases, malignant transformation to squamous cell carcinoma has been reported [3]. Antifungal therapy is usually effective but requires prolonged treatment (months to years) and is often too costly or unavailable in limited-resource settings [6].

## WHO’s road map for neglected tropical diseases 2021–2030

The WHO Road Map for NTDs 2021–2030 was released in 2020 and included chromoblastomycosis for the first time [10]. This road map provides disease-specific, cross-cutting targets, and overarching strategies to be achieved by 2030. The document also provides a gap assessment for each disease program along 11 dimensions which are divided under 3 broad categories: technical progress (scientific understanding; diagnostics; effective intervention), strategy and service delivery (operational and normative guidance; planning, governance and program implementation; monitoring and evaluation; access and logistics; health care infrastructure), and enablers (advocacy and funding; collaboration and multisectoral action; capacity and awareness building). The dimensions under these categories were assigned a gap assessment with 4 colors (green, yellow, orange, and red); green indicated no hindrance towards target while red indicated the most action needed to reach the target in the respective dimension. Chromoblastomycosis and other fungal infections, which included sporotrichosis and paracoccidioidomycosis, were included in the 2021–2030 roadmap. Chromoblastomycosis was designated as an NTD targeted for control, defined per WHO as reduction of disease incidence, prevalence, morbidity, and mortality to a locally or nationally acceptable level as a result of deliberate efforts. A WHO roadmap summary of the recommended critical actions for chromoblastomycosis, along with other implantation mycoses, were the establishment of surveillance, access to affordable diagnostics and treatment, creation of a field manual for diagnosing and treatment and proper training for health care workers, and development of a rapid diagnostic test and more effective treatment (S1 Table). All dimensions for chromoblastomycosis were assessed as orange (5/11) or red (6/11), indicating that critical action is required to reach the target goals.

To measure progress since the launch of the WHO Road Map for NTDs 2021–2030, an NTD gap assessment using a gap assessment tool was completed in December 2023 by an expert focus group for chromoblastomycosis focused on 4 key areas (diagnostics, access and logistics, advocacy and funding, and monitoring and evaluation). A similar color scheme was used in this gap assessment as the WHO roadmap. Each category retained the same original color status demonstrating that there had been no progress in the global objectives for the control of chromoblastomycosis since 2020.

## Development of global chromoblastomycosis working group

These findings led to the formation of a Global Chromoblastomycosis Working Group to advance the scientific and programmatic work on chromoblastomycosis globally. Initial virtual meetings were held in February, April, and May 2024 with over 20 combined individuals from 12 countries on 6 continents. Members include those from government, academia, and international organizations including WHO.

The working group developed a global strategy proposal to meet the 2030 targets set forth by the WHO roadmap. This proposed strategy provides an in-depth discussion and steps needed to achieve the actions in the WHO roadmap and added additional actions to help in the global control of chromoblastomycosis. The strategy follows the 11 specific dimensions laid out in the WHO roadmap (Table 1).

**Table 1:** Global chromoblastomycosis working group strategy on global control of chromoblastomycosis.

Category	Actions required
**Technical progress**	
Scientific understanding	• Better understanding of the role of melanin, muriform cells, and chitin in fungal virulence • Improve research on the impact of climate change (e.g., global warming, severe weather events) on causative fungi distribution and incidence • Better understand natural history and reservoirs of chromoblastomycosis-causing fungi • Evaluate risk factors and prevalence of chromoblastomycosis complications
Diagnostics	• Improve access to microscopes and training for direct mycological examination • Determine performance and feasibility of low-cost, practical sample collection methods like vinyl adhesive tape • Develop late diagnostic strategies, when muriform bodies or culture cannot be obtained, to detect refractory or relapsing infection • Develop epidemiological cutoff values for common causative fungi • Improve antifungal susceptibility testing practices • Increase research support to increase whole genome sequencing in endemic areas
Effective intervention	• Develop standardized treatment guidelines • Develop novel antifungals with less drug–drug interactions, side effects, absorption issues, and longer dosing intervals • Investigate other triazole antifungals (e.g., voriconazole, posaconazole) as initial and for refractory treatment • Assess itraconazole combination therapy with other pharmaceuticals, including topical antifungals, and non-pharmaceuticals
**Strategy and service delivery**	
Operational and normative guidance	• Develop comprehensive guidance on establishing chromoblastomycosis surveillance, prevention measures, and case management and control • Validate clinical staging system for chromoblastomycosis in different regions
Planning, governance, and program implantation	• Chromoblastomycosis activities should be incorporated into existing NTD infrastructure and programs
Monitoring and evaluation	• Develop global standardized case definitions and key surveillance and monitoring and evaluation indicators • Encourage countries to make chromoblastomycosis reportable nationally • Conduct precise mapping of causative fungi in endemic areas • Monitor for quality of life and disability burden
Access and logistics	• Improve sustainable access to both laboratory equipment and staining supplies • Increase continuity of access to itraconazole in rural and urban locations • Advocate for access or negotiated price to improve access to itraconazole
Health care infrastructure	• Strengthen primary health care for early detection, referrals, and follow-up care • Ensure access to health centers for wound management and WASH; integrate into skin NTD manual for wound management • Develop essential health care menu for integration into primary health care service essential care package
**Enablers**	
Advocacy and funding	• Encourage Ministry of Health and health leaders to make chromoblastomycosis a reportable disease and develop a national control program in the NTD and health sector plan • Pilot itraconazole donation programs to evaluate outcomes
Collaboration and multisectoral action	• Integration into existing NTD infrastructure at global, national, and subnational levels • Engagement with WASH activities and programs to raise awareness of chromoblastomycosis and evaluate WASH’s role in prevention • Clinicians, researchers, government and public health officials, patients, and policy makers can stay engaged with chromoblastomycosis through the Global Chromoblastomycosis Working Group
Capacity and awareness building	• Integrate chromoblastomycosis conventional diagnostic training with pan-fungal and skin-NTD diagnostic training • Develop chromoblastomycosis training for various levels of health care and disseminate the OpenWHO online course more widely

### Technical progress

#### Scientific understanding

Chromoblastomycosis is an implantation mycosis and is known to be acquired when fungi enter the skin after a traumatic transcutaneous injury. Melanized, filamentous fungi cause chromoblastomycosis and produce melanin in both reproductive and vegetative cells. Melanin may play a role in the virulence of chromoblastomycosis-causing fungi with potential mechanisms such as protection against proteolytic enzymes, protection against oxygen or nitrogen derivatives, or reduction of phagocytosis [9]. Melanin was shown to protect the transformation of hyphae and conidia into pigmented muriform bodies (sclerotic) with an increase in the thickness of the cell wall that provides a potentially resistance mechanism to host immune responses and antifungal drugs. Interestingly, melanin is extremely resistant to several physiochemical agents, including ultraviolet rays [11]. This phenomenon may help the chromoblastomycosis agents survive under the sun light in the environment. Chitin also plays a role in fungal structure and differentiation and may contribute to virulence and cell signaling [12]. Chromoblastomycosis can affect immunocompetent and immunosuppressed individuals, and better understanding the role of melanin, muriform cells, and chitin in fungal virulence can lead to novel therapeutic targets and clinical interventions. Certain genetic predisposing factors (e.g., HLA-A29, Card9) may interplay with melanin, muriform cells, and chitin for increased host-susceptibility and virulence and could be explored further to determine if infections may become more common in immunocompromised populations [13,14].

Most chromoblastomycosis-causing fungi have strong evidence to be climate-sensitive organisms [15]. Two of the most commonly reported genera have climate-niches, a finding supported by epidemiological data from Venezuela, Madagascar, and India; *F*. *pedrosoi* and *F*. *nubica* are commonly acquired in tropical regions while *C*. *carrionii* infections occur in more semiarid regions [4,16]. The impact of climate change could potentially restrict certain causative fungi while expanding niches for other fungi; further understanding of the impact of climate change is warranted. Increased severe weather events (e.g., hurricanes, flooding) from climate change can impact rates of chromoblastomycosis, and this association should be further studied for potential public health interventions [17,18].

In addition to climate change’s impact on chromoblastomycosis-causing fungi, further understanding of the natural history and reservoirs of these fungi can inform prevention and awareness raising efforts. Previous chromoblastomycosis infections or causative fungi have been associated with certain plants (e.g., thorns of *Mimosa pudica*, several species of the *Palmacea* family including Madagascar palm house plant, Jurubeba, Murta tree, Tucum tree, Vassourinha tree, Bacuri tree, and babassu coconut, and hydrocarbon-polluted environments such as wood treated with phenolic preservatives, toxic mine waste, oil-polluted soils) [19–24]. Even some injuries caused by animals like birds, insects’ stings, and snakes have been associated as the port of entry in some patients [9,19]. Some molecular environmental tests and metagenomics of soils and plants could be used to overcome culture drawbacks (e.g., difficult to isolate from environment) to elucidate differences in reservoirs of various chromoblastomycosis-causing fungi. Whole genome sequencing, if cultures are obtained, can help establish links between environmental isolates, ecological niches, and human infections [25,26].

Chromoblastomycosis can lead to tissue fibrosis, lymphedema, secondary bacterial infections, squamous cell carcinoma, ankylosis, ectropium, and other serious and debilitating complications. [9] Some of these complications seem to be associated with illness duration, severity of traumatic injury, and vegetating lesions; however, risk factors for and the overall magnitude of chromoblastomycosis complications are relatively unknown despite their severity [27,28].

#### Diagnostics

Diagnosis of chromoblastomycosis depends heavily on clinical expertise and access to direct mycological examination (e.g., potassium hydroxide preparation) or histopathology. Identification of muriform bodies provides a confirmed diagnosis, but it is dependent on obtaining a sample from a correct location on the lesion and having materials and technical expertise in microscopy [29]. With proper training, even in resource-limited settings, direct microscopy can elucidate a diagnosis with high sensitivity and can be a high-yield, low-cost public health intervention [7]. Dermoscopy may be able to improve identification of the most suitable locations (i.e., black dots within the verrucous plaques) on lesions to sample, but equipment is expensive, and its use has not been validated thoroughly [30]. A practical and low-cost method for collecting samples in the field could be the use of vinyl adhesive tape; this tape could be subsequently used for direct microscopy [31]. Development of a universally available, affordable method for easy visualization of muriform bodies could improve early identification of chromoblastomycosis and allow primary health care to diagnose and treat infections. Late diagnostic strategies, when muriform bodies or culture cannot be obtained, to detect refractory or relapsing infection after months of therapy are critical to develop to prevent further complications and quality of life problems. Previous research has been completed on the potential of serologic diagnosis and could be explored further to help establish a diagnosis of chromoblastomycosis and inform prevalence studies [32,33].

Antifungal resistance continues to emerge globally in a variety of fungi. Antifungal susceptibility testing (AFST) on chromoblastomycosis-causing fungi is rarely performed as the technology is often unavailable in areas where chromoblastomycosis occurs; however, the development of epidemiolocal cutoff values for common causative fungi can help monitor for rising minimum inhibitory concentrations (MICs) and potential treatment failure. One study demonstrated that MICs of sequential isolates of *F*. *pedrosoi* increased over time during treatment and may have contributed to microbiological resistance to itraconazole with some isolates resulting in a lack of clinical response [34]. Increased AFST can provide clues into reasons for treatment failure and inform change of therapy. Cultures may be difficult to obtain routinely from the slow growth of fungi and inaccessibility to culture supplies; programs to routinely perform surveillance on DNA to identify resistant genetic markers for causative fungi would improve clinical care and inform public health interventions.

#### Effective intervention

Treatment of chromoblastomycosis can be challenging, particularly in moderate and severe disease which requires long duration of systemic antifungal therapy (e.g., minimum of 6 months). If diagnosed early, surgical excision can be effective, but usually also requires the use of antifungals. The development of standardized treatment guidelines may improve treatment practices despite a lack of randomized clinical trials. Itraconazole is typically noted as the initial therapeutic choice for chromoblastomycosis, but cure rates range from 15% to 80% [27,35–37]. In addition to low cure rates, itraconazole has drug–drug interactions and side effects, and itraconazole absorption is dependent on gastric acidity. Novel antifungals that have less pharmacokinetic variation and longer dosing windows can improve treatment outcomes. A recent and first clinical trial for eumycetoma, another fungal NTD, included a novel antifungal, fosravuconazole, and showed promising results. Fosravuconazole is absorbed better and has once weekly dosing; this antifungal could also be useful in the treatment of chromoblastomycosis but further research is needed [38]. Likewise, work on the use of melanin biosynthesis inhibitors in experimental models of mycetoma may also provide a lead to new treatment strategies in chromoblastomycosis [38]. Combination therapy with other pharmaceuticals, including topical antifungals, and non-pharmaceutical therapies could improve treatment success, but current knowledge is largely based on case reports. Potential combinations with itraconazole include surgical excision, immunomodulators like imiquimod, cryotherapy, terbinafine, and flucytosine [39–43]. Other triazole antifungals (e.g., voriconazole, posaconazole) may play a role in refractory disease, but these are often unavailable or unaffordable in regions where chromoblastomycosis occurs [6,44]. Clinicians and researchers who are utilizing these newer antifungals or combination therapies can submit case data to open-access platforms like CURE ID which can collate treatment and outcome data globally and make the data publicly available [45]. Finally, improved healthcare service delivery to ensure the provision of effective medicines early in the disease process and with no or low cost to the patient must be prioritized.

Prevention interventions for chromoblastomycosis have not been extensively evaluated, although, personal protective equipment (e.g., gloves, shoes, appropriate clothing) and water, sanitation and hygiene (WASH) have been proposed as public health interventions. Specific prevention research can provide evidence for certain interventions, and these data can be used to engage local and global partners to prevent disease. Improvements in knowledge among frontline health workers and earlier recognition of chromoblastomycosis can help prevent severe disease in addition to increase access to appropriate diagnostics and treatment and improved follow-up through local district facility networks (e.g., lab technicians, clinicians).

### Strategy and service delivery

#### Operational and normative guidance

Other logistic interventions are needed to improve patient care. For example, comprehensive guidance on establishing chromoblastomycosis surveillance (including universally accepted case definitions and monitoring and evaluation indicators), prevention measures, and case management and control can encourage national programs for chromoblastomycosis. This guidance can be integrated with other skin-NTDs as described below. From a clinical perspective, a clinical staging system for disease has been proposed and can be validated further in multiple regions [9].

#### Planning, governance, and program implementation

While chromoblastomycosis is a distinct implantation mycosis that differs clinically and epidemiologically from mycetoma, sporotrichosis, and other non-fungal NTDs. Progress against this NTD can likely be best achieved by incorporating chromoblastomycosis in cross-cutting NTD programs at national and subnational levels, particularly in the context of universal health coverage.

#### Monitoring and evaluation

Current estimates of chromoblastomycosis burden and distribution are based on randomly collected and published case reports [3]. In the 2024 WHO Global Report on Neglected Tropical Diseases, only Madagascar submitted national data on chromoblastomycosis [46]. Countries can prioritize making chromoblastomycosis a nationally reportable disease to improve the understanding of the true incidence and prevalence. Global health entities and chromoblastomycosis experts can develop standardized case definitions and surveillance indicators to provide uniform data across regions. Quantitative quality of life and disability data have not been reported for chromoblastomycosis but could be adapted easily using currently available scales and scoring systems (e., Dermatology Quality Life Index); studies evaluating these morbidities can inform the true burden of chromoblastomycosis. Taken together with the assessment of the economic impact of the disease on families would provide much needed data on the true cost of chromoblastomycosis in poor communities.

#### Access and logistics

**Fig 1:**
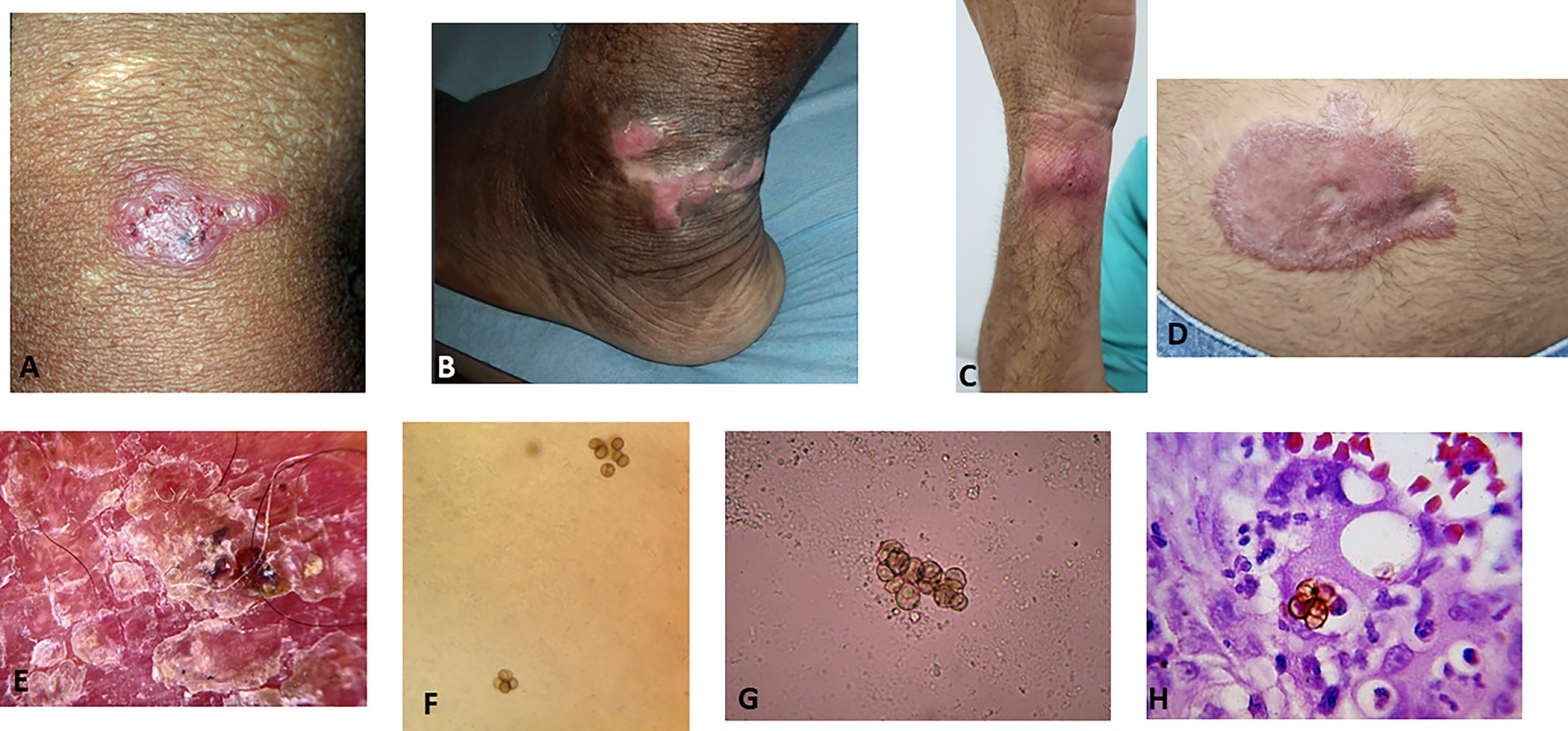
Early chromoblastomycosis lesions and histopathological and microbiological images. (A–D) Early chromoblastomycosis lesions presenting as erythematous macules and papules on the legs, arm, and stomach. (E) Visualization using dermoscopy showing erythema, scales, and blackheads. (F, G) Direct microscopy using potassium hydroxide wet mount showing muriform cells. (H) Hematoxylin and eosin stain showing muriform cells.

Access to conventional diagnostics is limited in many countries, particularly noted in more rural areas [6,47,48]. Improving sustainable access to both laboratory equipment (e.g., microscopes, slides, culture plates and media) and supplies of stains and other laboratory reagents will be critical for early diagnosis and prevention of severe disease (Fig 1). Increased access to diagnostics will need to be partnered with increased access to affordable, safe, and effective antifungal drugs such as itraconazole; at least 78 million people have no access to this antifungal [49]. Itraconazole is widely available in Brazil, free of charge, for implantation mycoses including chromoblastomycosis. Itraconazole is distributed through a special program from the Brazilian Ministry of Health to all patients treated in the Public Health System. Similar programs can be developed in other endemic countries. Various formulations and generic forms of itraconazole are widely available in certain regions of the world (e.g., South and Southeast Asia), but need to meet quality standards including acceptable pharmacokinetic measures. International efforts need to be made to supply low and middle income countries with affordable itraconazole.

#### Health care infrastructure and workforce

By strengthening and funding primary health care infrastructure, and building a sustainable workforce, chromoblastomycosis can be detected earlier and managed more appropriately using simple educational tools from courses, online teaching programs, and diagnostic applications utilizing artificial intelligence. Referral systems can be established for higher level of diagnosis and care for refractory disease. Access to primary health care can provide wound care and education on the importance of WASH to healthcare workers and the public.

### Enablers

#### Advocacy and funding

Clinicians and researchers should discuss the addition of chromoblastomycosis as a reportable disease with their Ministry of Health and national health leaders and include in cross-cutting NTD activities. In highly endemic areas, clinicians and public health officials can connect with pharmaceutical companies manufacturing itraconazole and conduct pilot studies with donated medication to describe treatment outcomes. NTD advocate groups, professional societies, and the international community should include chromoblastomycosis and other implantation mycoses in their advocacy strategy and events. These pilot projects may lead to larger, consistent drug donations or reduction in prices to control chromoblastomycosis globally.

#### Collaboration and multisectoral action

Integration into existing NTD infrastructure should be prioritized to improve the control of chromoblastomycosis at global, national, and subnational levels. In particular, this disease has a low profile in current global initiatives of integrated skin-NTD pathways. Engagement with WASH activities and programs could provide additional venues to raise awareness of chromoblastomycosis and evaluate WASH’s role in prevention (e.g., personal hygiene, access to quality water). Clinicians, researchers, government and public health officials, patients, and policy makers can stay engaged with chromoblastomycosis through the Global Chromoblastomycosis Working Group.

#### Capacity and awareness building

Conventional diagnostic training for chromoblastomycosis can be integrated in pan-fungal disease diagnostic and pan-NTD diagnostic training for both clinicians and laboratorians. Frontline healthcare and laboratory workers would be key targets for chromoblastomycosis diagnostic training, but appropriate training materials are needed for various health care cadres including dermatologists and infectious diseases physicians. Current training platforms, like the OpenWHO frontline health worker training on chromoblastomycosis, can be disseminated, built upon, and translated into additional languages [50]. Training should include education on avoiding the use of topical or systemic corticosteroids for itching or burning associated with chromoblastomycosis as these products can extend and complicate lesions. Community-based organizations can be engaged to raise awareness of chromoblastomycosis among the public and encourage them to seek care for painless lesions.

#### Joining the Global Chromoblastomycosis Working Group

The Global Chromoblastomycosis Working Group is committed to following this strategy to improve the knowledge and care of chromoblastomycosis. Clinicians, researchers, public health and government officials, nongovernmental organizations, patients, and policy makers are encouraged to join this working group to contribute their expertise. Interested individuals can sign-up using this link: https://forms.gle/jT7SSFNEgHGPZtDX9.

## Conclusion

Improving control of chromoblastomycosis can be achieved through this global strategy outlined and proposed by the Global Chromoblastomycosis Working Group. Currently available diagnostic tools and therapeutics can be effective if access is expanded to areas where chromoblastomycosis is common; use of diagnostics and therapeutics can improve the epidemiological understanding of this NTD. Progress in pathophysiology knowledge, novel diagnostics and treatment, and global and local partnerships can occur simultaneously to improve care for patients. Through this global chromoblastomycosis strategy, clinicians, researchers, public and government officials, patients, and policy makers can align time, expertise, and resources to improve the lives of communities affected by chromoblastomycosis.

### Disclaimer

The findings and conclusions in this report are those of the authors and do not necessarily represent the official position of the US Centers for Disease Control and Prevention and the World Health Organization.

## Supporting information

S1 Table World Health Organization Road Map for Neglected Tropical Diseases 2021–2030: Chromoblastomycosis Category and Actions Required*. (DOCX)

## References

[1] World Health Organization. Neglected Tropical Diseases. Available from: https://www.who.int/health-topics/neglected-tropical-diseases#tab=tab_1.

[2] Queiroz-Telles F, Nucci M, Colombo AL, Tobón A, Restrepo A. Mycoses of implantation in Latin America: an overview of epidemiology, clinical manifestations, diagnosis and treatment. Med Mycol. 2011;49(3):225–236. doi: 10.3109/13693786.2010.539631 21128710

[3] Santos DWCL, de Azevedo CMPES, Vicente VA, Queiroz-Telles F, Rodrigues MA, et al. The global burden of chromoblastomycosis. Hay R, ed. PLoS Negl Trop Dis. 2021;15(8):e0009611. doi: 10.1371/journal.pntd.0009611 34383752 PMC8360387

[4] Rasamoelina T, Maubon D, Andrianarison M, et al. Endemic Chromoblastomycosis Caused Predominantly by *Fonsecaea nubica*, Madagascar1. Emerg Infect Dis. 2020;26(6):1201–1211. doi: 10.3201/eid2606.191498 32441639 PMC7258462

[5] Bongomin F, Gago S, Oladele R, Denning D. Global and Multi-National Prevalence of Fungal Diseases—Estimate Precision. J Fungi. 2017;3(4):57. doi: 10.3390/jof3040057 29371573 PMC5753159

[6] Milani B, Dagne DA, Choi HL, Schito M, Stone HA. Diagnostic capacities and treatment practices on implantation mycoses: Results from the 2022 WHO global online survey. Nosanchuk J, editor. PLoS Negl Trop Dis. 2023;17(6):e0011443. doi: 10.1371/journal.pntd.0011443 37379338 PMC10335693

[7] Santos DWCL Vicente VA, Weiss VA, et al. Chromoblastomycosis in an Endemic Area of Brazil: A Clinical-Epidemiological Analysis and a Worldwide Haplotype Network. J Fungi. 2020;6(4):204. doi: 10.3390/jof6040204 33022951 PMC7711792

[8] Bombassaro A, Schneider GX, Costa FF, et al. Genomics and Virulence of *Fonsecaea pugnacius*, Agent of Disseminated Chromoblastomycosis. Front Genet. 2020;11:822. doi: 10.3389/fgene.2020.00822 32849816 PMC7417343

[9] Queiroz-Telles F, de Hoog S, Santos DWCL, et al. Chromoblastomycosis. Clin Microbiol Rev. 2017;30(1):233–276. doi: 10.1128/CMR.00032-16 27856522 PMC5217794

[10] World Health Organization. Ending the neglect to attain the Sustainable Development Goals: A road map for neglected tropical diseases 2021–2030. 2021 January 28. Accessed 2024 May 5. Available from: https://www.who.int/publications/i/item/9789240010352.

[11] Dadachova E, Casadevall A. Ionizing radiation: how fungi cope, adapt, and exploit with the help of melanin. Curr Opin Microbiol. 2008;11(6):525–531. doi: 10.1016/j.mib.2008.09.013 18848901 PMC2677413

[12] Lenardon MD, Munro CA, Gow NA. Chitin synthesis and fungal pathogenesis. Curr Opin Microbiol. 2010;13(4):416–423. doi: 10.1016/j.mib.2010.05.002 20561815 PMC2923753

[13] Tsuneto LT, Arce-Gomez B, Petzl-Erler ML, Queiroz-Telles F. HLA-A29 and genetic susceptibility to chromoblastomycosis. Med Mycol. 1989;27(3):181–185. doi: 10.1080/02681218980000241 2778577

[14] Sobianski Herman T, de Azevedo CMPES, Lorenzetti Bocca A. CARD9 mutations and T cell immune response in patients with chromoblastomycosis. One Health Mycol. 2024;1(1):14–22. doi: 10.63049/OHM.24.11.3

[15] Bonifaz A, Robles-Tenorio A, Tirado-Sánchez A. Climate Change Impact on Chromoblastomycosis. In: Frías-De-León MG, Brunner-Mendoza C, Reyes-Montes M del R, Duarte-Escalante E, editors. The Impact of Climate Change on Fungal Diseases. Fungal Biology. Springer International Publishing; 2022. p. 115–129. doi: 10.1007/978-3-030-89664-5_7

[16] Gadre A, Enbiale W, Andersen LK, Coates SJ. The effects of climate change on fungal diseases with cutaneous manifestations: A report from the International Society of Dermatology Climate Change Committee. J Clim Change Health. 2022;6:100156. doi: 10.1016/j.joclim.2022.100156

[17] Riddel CE, Surovik JG, Chon SY, et al. Fungal foes: presentations of chromoblastomycosis post-hurricane Ike. Cutis. 2011;87(6):269–272. 21838080

[18] Bandino JP, Hang A, Norton SA. The Infectious and Noninfectious Dermatological Consequences of Flooding: A Field Manual for the Responding Provider. Am J Clin Dermatol. 2015;16(5):399–424. doi: 10.1007/s40257-015-0138-4 26159354

[19] Brenes H, Herrera ML, Ávila-Aguero ML. Chromoblastomycosis Caused by *Phialophora verrucosa* in a Costa Rican Child with Skin Sequelae due to Snake Bite. Cureus. Published online November 12, 2018. doi: 10.7759/cureus.3574 30656078 PMC6333267

[20] Vicente VA, Attili-Angelis D, Pie MR, et al. Environmental isolation of black yeast-like fungi involved in human infection. Stud Mycol. 2008;61:137–144. doi: 10.3114/sim.2008.61.14 19287536 PMC2610314

[21] Salgado CG, da Silva JP Diniz JAP, et al. Isolation of *Fonsecaea pedrosoi* from thorns of Mimosa pudica, a probable natural source of chromoblastomycosis. Rev Inst Med Trop São Paulo. 2004;46(1):33–36. doi: 10.1590/s0036-46652004000100006 15057332

[22] Smith G, Chen AF, Weiss E. Chromoblastomycosis infection from a house plant. Cutis. 2017;100(4):E13–E14. 29136064

[23] Voidaleski MF, Gomes RR, de Azevedo CMPES, de Souza Lima BJF, de Fátima Costa F, et al. Environmental Screening of *Fonsecaea* Agents of Chromoblastomycosis Using Rolling Circle Amplification. J Fungi. 2020;6(4):290. doi: 10.3390/jof6040290 33212756 PMC7712894

[24] Vicente VA, Najafzadeh MJ, Sun J, et al. Environmental siblings of black agents of human chromoblastomycosis. Fungal Divers. 2014;65(1):47–63. doi: 10.1007/s13225-013-0246-5

[25] Costa F de F, Souza RC, Voidaleski MF, Bombassaro A, Candido GZ, da Silva NM, et al. New Insights on Environmental Occurrence of Pathogenic Fungi Based on Metagenomic Data from Brazilian Cerrado Biome. Braz Arch Biol Technol. 2022;65:e22210097. doi: 10.1590/1678-4324-2022210097

[26] Voidaleski MF, Costa F de F, de Hoog GS, Gomes RR, Vicente VA. Metagenomics reveals an abundance of black yeast-like fungi in the skin microbiome. Mycoses. 2023;66(6):488–496. doi: 10.1111/myc.13574 36740746

[27] Bonifaz A, Carrasco-Gerard E, Saúl A. Chromoblastomycosis: clinical and mycologic experience of 51 cases. Mycoses. 2001;44(1–2):1–7. doi: 10.1046/j.1439-0507.2001.00613.x 11398635

[28] de Azevedo CMPES Marques SG, Santos DWCL, et al. Squamous Cell Carcinoma Derived From Chronic Chromoblastomycosis in Brazil. Clin Infect Dis. 2015;60(10):1500–1504. doi: 10.1093/cid/civ104 25681378

[29] Wanat KA, Dominguez AR, Carter Z, Legua P, Bustamante B, Micheletti RG. Bedside diagnostics in dermatology. J Am Acad Dermatol. 2017;77(2):197–218. doi: 10.1016/j.jaad.2016.06.034 28711082

[30] Chauhan P, Meena D, Errichetti E. Dermoscopy of Bacterial, Viral, and Fungal Skin Infections: A Systematic Review of the Literature. Dermatol Ther. 2023;13(1):51–76. doi: 10.1007/s13555-022-00855-2 36417086 PMC9823193

[31] Miranda MFR, Silva AJG. Vinyl adhesive tape also effective for direct microscopy diagnosis of chromomycosis, lobomycosis, and paracoccidioidomycosis. Diagn Microbiol Infect Dis. 2005;52(1):39–43. doi: 10.1016/j.diagmicrobio.2005.02.008 15878441

[32] Vidal MSM, Castro LGM, Cavalcante SC, Lacaz CS. Highly specific and sensitive, immunoblot-detected 54 kDa antigen from *Fonsecaea pedrosoi*. Med Mycol. 2004;42(6):511–515. doi: 10.1080/13693780310001654337 15682639

[33] Esterre P, Jahevitra M, Andriantsimahavandy A. Humoral Immune Response in Chromoblastomycosis during and after Therapy. Clin Diagn Lab Immunol. 2000;7(3):497–500. doi: 10.1128/CDLI.7.3.497-500.2000 10799467 PMC95900

[34] Andrade TS, Castro LGM, Nunes RS, Gimenes VMF, Cury AE. Susceptibility of sequential *Fonsecaea pedrosoi* isolates from chromoblastomycosis patients to antifungal agents. Mycoses. 2004;47(5–6):216–221. doi: 10.1111/j.1439-0507.2004.00984.x 15189187

[35] Queiroz-Telles F, Purim KS, Fillus JN, et al. Itraconazole in the treament of chromoblastomycosis due to *Fonsecaea pedrosoi*. Int J Dermatol. 1992;31(11):805–812. doi: 10.1111/j.1365-4362.1992.tb04252.x 1330949

[36] Restrepo A, Gonzalez A, Gomez I, Arango M, Bedout CD. Treatment of Chromoblastomycosis with Itraconazole. Ann N Y Acad Sci. 1988;544(1):504–516. doi: 10.1111/j.1749-6632.1988.tb40448.x 2850755

[37] Sendrasoa FA, Ratovonjanahary VT, Rasamoelina T, Ramarozatovo LS, Rapelanoro Rabenja F. Treatment responses in patients with chromoblastomycosis to itraconazole in Madagascar. Med Mycol. 2022;60(11):myac086. doi: 10.1093/mmy/myac086 36288247

[38] Chandler DJ, Bonifaz A, van de Sande WWJ. An update on the development of novel antifungal agents for eumycetoma. Front Pharmacol. 2023;14:1165273. doi: 10.3389/fphar.2023.1165273 37274106 PMC10232793

[39] Logan C, Singh M, Fox N, et al. Chromoblastomycosis treated with posaconazole and adjunctive imiquimod: lending innate immunity a helping hand. Open Forum Infect Dis. Published online March 14, 2023:ofad124. doi: 10.1093/ofid/ofad124 37035498 PMC10077821

[40] Rolon AM, Tolaymat LM, Sokumbi O, Bodiford K. The Role of Excision for Treatment of Chromoblastomycosis: A Cutaneous Fungal Infection Frequently Mistaken for Squamous Cell Carcinoma. Dermatol Surg. 2023;Publish Ahead of Print. doi: 10.1097/DSS.0000000000003800 37093678

[41] Bonifaz A, Martínez-Soto E, Carrasco-Gerard E, Peniche J. Treatment of chromoblastomycosis with itraconazole, cryosurgery, and a combination of both. Int J Dermatol. 1997;36(7):542–547. doi: 10.1046/j.1365-4362.1997.00085.x 9268758

[42] Antonello VS, da Silva MCA, Cambruzzi, Kliemann DA, Santos BR, Queiroz-Telles F. Treatment of severe chromoblastomycosis with itraconazole and 5-flucytosine association. Rev Inst Med Trop São Paulo. 2010;52(6):329–331. doi: 10.1590/s0036-46652010000600008 21225217

[43] Gupta AK, Taborda PR, Sanzovo AD. Alternate week and combination itraconazole and terbinafine therapy for chromoblastomycosis caused by *Fonsecaea pedrosoi* in Brazil. Med Mycol. 2002;40(5):529–534. doi: 10.1080/mmy.40.5.529.534 12462534

[44] Negroni R, Tobón A, Bustamante B, Shikanai-Yasuda MA, Patino H, Restrepo A. Posaconazole treatment of refractory eumycetoma and chromoblastomycosis. Rev Inst Med Trop São Paulo. 2005;47(6):339–346. doi: 10.1590/s0036-46652005000600006 16553324

[45] CURE ID. Available from: https://cure.ncats.io/about. Accessed 2024 May 8.

[46] Global report on neglected tropical diseases 2024. Geneva: World Health Organization; 2024.

[47] Badiane AS, Ramarozatovo LS, Doumbo SN, et al. Diagnostic capacity for cutaneous fungal diseases in the African continent. Int J Dermatol. 2023;62(9):1131–1141. doi: 10.1111/ijd.16751 37340531

[48] Salmanton-García J, Au WY, Hoenigl M, et al. The current state of laboratory mycology in Asia/Pacific: A survey from the European Confederation of Medical Mycology (ECMM) and International Society for Human and Animal Mycology (ISHAM). Int J Antimicrob Agents. Published online January 2023:106718. doi: 10.1016/j.ijantimicag.2023.106718 36640851

[49] Kneale M, Bartholomew JS, Davies E, Denning DW. Global access to antifungal therapy and its variable cost. J Antimicrob Chemother. 2016;71(12):3599–3606. doi: 10.1093/jac/dkw325 27516477

[50] World Health Organization. Chromoblastomycosis: Training for National and District-level Health Workers. OpenWHO. Available from: https://openwho.org/courses/NTDs-chromoblastomycosis.

