# Supplementary material for: A global chromoblastomycosis strategy and development of the global chromoblastomycosis working group
Source: PLoS Negl Trop Dis. 2024 Oct 15;18(10):e0012562. doi: 10.1371/journal.pntd.0012562 (PMC11478817; doi:10.1371/journal.pntd.0012562)
Supplement: S1 Table — (DOCX) [file pntd.0012562.s001.docx]

**S1 Table: World Health Organization Road Map for Neglected Tropical Diseases 2021–2030: Chromoblastomycosis Category and Actions Required***

| **Category** | **Actions required** |
| --- | --- |
| **Technical progress** | |
| Scientific understanding | - Determine the exact magnitude, trend and distribution of the diseases and associated causative agents |
| Diagnostics | - Develop rapid diagnostic or serological tests to improve early detection at primary health care level - Facilitate skin scarping and biopsy, and fungal culture and histopathology assessment of deep skin lesions |
| Effective intervention | - Evaluate effectiveness of itraconazole and other antifungals through prospective studies - Improve therapeutic regimens (shorter duration and increased efficacy) to increase responsiveness to treatment) - Develop innovative preventive tools based on local understanding of the transmission |
| **Strategy and service delivery** | |
| Operational and normative guidance | - Develop grading system to assess clinical stage, valid for all endemic regions - Develop field manual on prevention, case management, and surveillance |
| Planning, governance and program implantation | - Include in control programs against NTDs or communicable diseases in endemic countries |
| Monitoring and evaluation | - Develop a surveillance guide with standard indicators - Establish surveillance system for active case detection - Establish M&E system or integrate with national health information system |
| Access and logistics | - Ensure access to affordable and quality-assured itraconazole - Provide affordable skin biopsy and sample processing for diagnosis |
| Health care infrastructure | - Strengthen the health system to provide care within context of universal health coverage - Ensure sufficient laboratory capacity for appropriate and timely diagnosis |
| **Enablers** | |
| Advocacy and funding | - Ensure political commitment from endemic countries and partners to mobilize funds and human resources - Engage community and mobilize support for programme implementation |
| Collaboration and multisectoral action | - Initiate collaboration with various research institutes, medicines and diagnostics developers, manufacturers and donors to improve access to diagnosis and treatment in all endemic countries - Coordinate with WASH stakeholders to ensure improved water supplies and soap availability in households and health care facilities |
| Capacity and awareness building | - Train health professionals and community health workers across priority skin NTDs to improve early detection based on local epidemiological contexts - Improve the diagnostic (e.g. skin biopsy and histopathology) and managing capacities of health care system in the endemic regions of the countries |

*Modified from: World Health Organization. Ending the neglect to attain the Sustainable Development Goals: A road map for neglected tropical diseases 2021–2030.
